# Impact of Lower Limb Active Movement Training in Individuals With Spastic Type Cerebral Palsy on Neuromuscular Control Outcomes: A Systematic Review

**DOI:** 10.3389/fneur.2020.581892

**Published:** 2020-11-26

**Authors:** Shari M. O'Brien, Glen A. Lichtwark, Timothy J. Carroll, Lee A. Barber

**Affiliations:** ^1^School of Human Movement and Nutrition Sciences, Faculty of Health and Behavioural Sciences, The University of Queensland, Brisbane, QLD, Australia; ^2^Centre for Sensorimotor Performance, The University of Queensland, Brisbane, QLD, Australia; ^3^School of Allied Health Sciences, Griffith University, Brisbane, QLD, Australia; ^4^Child Health Research Centre, Faculty of Medicine, The University of Queensland, Brisbane, QLD, Australia

**Keywords:** cerebral palsy, lower limb, intervention, motor control, voluntary activation

## Abstract

**Background:** Cerebral Palsy (CP) is a non-progressive neurological condition that results in motor impairment which increases proximally to distally along the lower extremity (i.e., greatest impairment at the ankle). Consequently, motor impairment and reduced voluntary muscle activation results in reduced neuromuscular control of the lower limb in this population. CP rehabilitation traditionally aims to improve movement proficiency for functional activities, such as walking, by using a range of active movement modalities that require volitional effort; however, the underlying neural mechanisms of improved control and function remain unknown. The primary purpose of this study was to systematically determine the efficacy of lower limb active movement interventions to improve neuromuscular control in individuals with CP.

**Methodology:** A search for studies involving an active lower limb intervention and neurophysiological outcome measures in individuals with CP was performed in five electronic databases. Studies were assessed for methodological quality using the Downs and Black assessment tool.

**Results:** Nine of 6,263 articles met the inclusion criteria. Methodological quality of all studies was poor, ranging from 2 to 27 out of a possible score of 32 points on the Downs and Black assessment tool. The study interventions varied extensively in modality and prescription as well as in the outcome measures used.

**Conclusions:** Whether active movement improves neuromuscular control of the lower limb in CP is unclear due to high variability in intervention protocols and selected outcomes measures. Future active intervention studies must carefully consider the selection of neurophysiological outcome measures.

## Introduction

Spastic type Cerebral Palsy (CP) is a motor impairment syndrome resulting from a non-progressive perinatal brain lesion. It is a lifelong condition which reduces an individual's ability to control posture and bodily movements (1). Following a central nervous system lesion, changes in development of the descending pathways causes a reduction in voluntary muscle activation and increased tonic muscle activity which may include spasticity, dystonia, hypertonia, and co-contraction (2–4). Within the lower limb, distal muscles are more impaired (5), which directly impacts lower limb neuromuscular control and the capacity to perform activities of daily living requiring ambulation (6, 7).

Lower limb functional activities of daily living such as ambulation, standing from a chair and climbing stairs, all require control over limb movements to achieve the specific task. To investigate the neuromuscular control required to perform these tasks, and the potential for neuroplasticity following motor learning, both the nervous system and the movement must be considered. Common clinical measures of CP lower limb functional capacity assess gross movement, but do not directly measure neural function during these gross motor tasks. For example, the 6 min walk test measures distance traveled (8), while the selective control assessment of the lower limb rates isolated joint movements (9). Neither assessments involve concurrent measures which probe the nervous system, for example recordings of electrical muscle activity or neural re-organization. To understand underlying neural mechanisms, neurophysiological assessments of the central and peripheral nervous systems are required. Neurophysiological assessment methods including electromyography (EMG) and magnetic resonance imaging (MRI) can determine muscle activation capacity, motor unit firing patterns and firing frequency as well as the amplitude of interference EMG signals and neural tract organization (2, 10, 11). Cross-sectional studies in individuals with CP report reduced capacity to voluntarily activate the plantar flexors and quadriceps by 20–50% (2, 4), reduced electromyography amplitude and motor unit firing rates (12), and altered muscle activation patterns, particularly for distal agonists (13–16).

Active movement training will herein be defined as an intervention requiring volitional muscular effort to achieve a given movement task, exclusive of assisted or passive movements. Intervention studies which aim to improve lower limb joint function, muscular impairment, and functional activity in individuals with CP often use active training modalities including treadmill walking and resistance training which achieve clinically important improvements in strength and walking ability, but typically do not assess neurophysiological outcomes which may indicate neuroplasticity following a perinatal brain lesion (17–19).

Increases in strength achieved with resistance training indicate the trainability of lower limb musculature in CP (20, 21). As knowledge of the neural mechanisms of motor impairment in CP is limited, understanding the neural component of movement control and its adaptability in CP is of great importance for rehabilitation. Preliminary evidence of enhanced neuromuscular control following lower limb active movement training exists in healthy adults and individuals with stroke (22–25), however few studies have used neurophysiological measures to assess changes in neural function and neuromuscular control following interventions in CP. The purpose of this study was to systematically review the current literature to determine the impact of lower limb active movement training in CP on neuromuscular control.

## Survey Methodology

### Search Strategy

**Figure 1:**
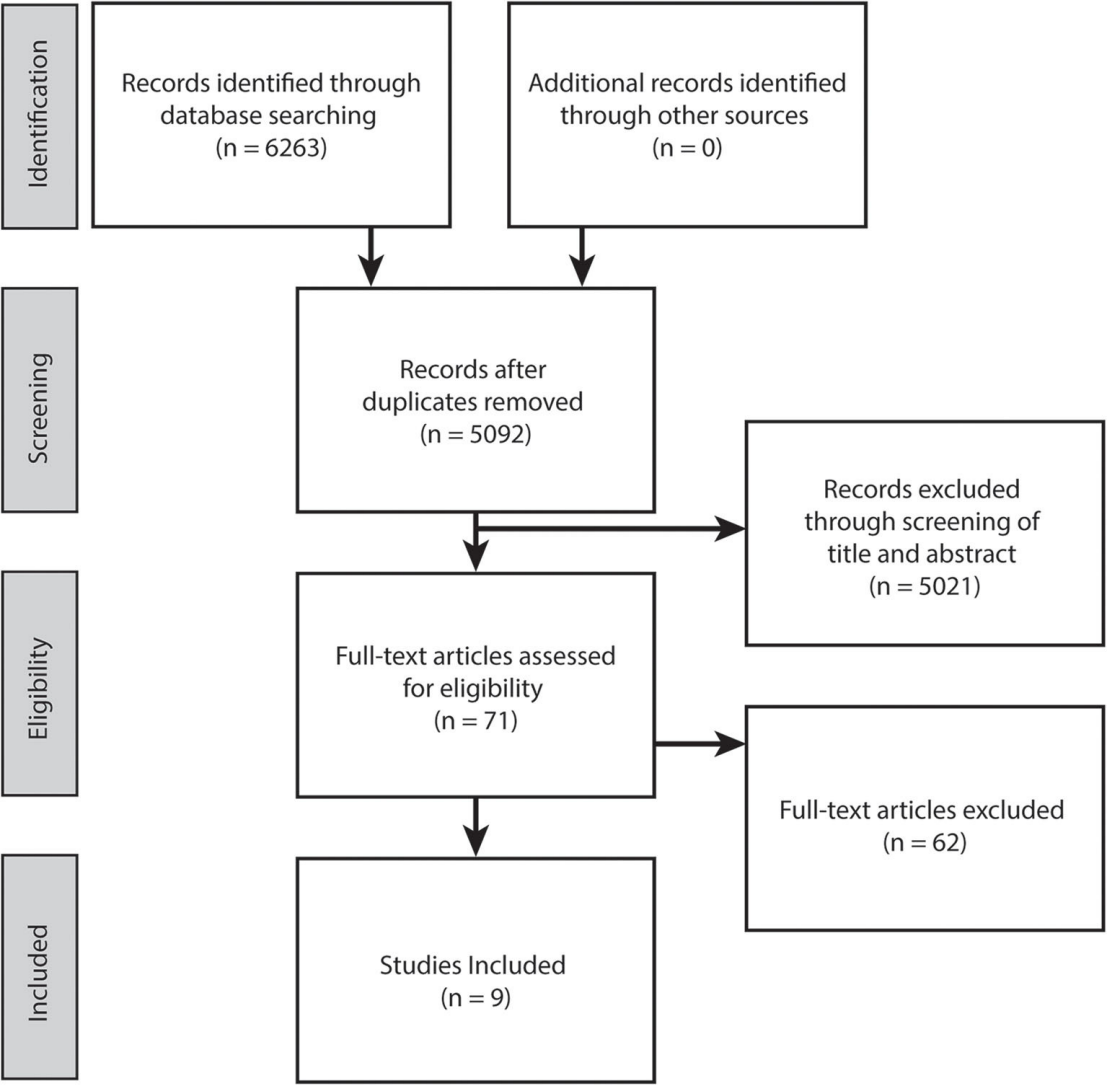
PRISMA flow diagram.

This review was conducted following PRISMA guidelines (Figure 1). An electronic literature search was conducted in September, 2020 within five online databases (Pubmed, CINAHL, Cochrane library, Embase and PEDro). The search strategy comprised of the following keywords: (i) population: (cerebral palsy) AND (lower limb or lower extremity or leg or hip or knee or ankle) AND (ii) intervention: (exercise or training or trial or active movement or rehabilitation or walk^*^ or game^*^ or robot^*^ or therapy or resistance or isometric or isotonic or isokinetic or aerobic or anaerobic) AND (iii) outcome: (neuromuscular or neuromotor or motor control or selective motor control or voluntary activation or volition^*^ or interpolated twitch or electromyography or rate of force development or function or gait or control or coordination or gait analysis or motion analysis). Note the PEDro database keyword search was reduced to “cerebral palsy motor control.” Article full texts that were not available electronically were retrieved through the University of Queensland Library document delivery service.

### Inclusion Criteria

Articles were screened via title and abstract by the first author (SO'B) and full texts were retrieved when eligibility could not be established from the abstract. Articles were included if they satisfied the following criteria: (i) ambulatory individuals of any age diagnosed with cerebral palsy, (ii) comprised of an active movement intervention including the lower limb which required volitional muscular effort of the paretic limb/s (any inclusion of passive modalities i.e., botulinum toxin, massage, passive range of motion, stretching, orthosis, surgery, motor driven robotics, and electrical stimulation resulted in exclusion), (iii) reported neurophysiological outcome measures (using electromyography or neuroimaging techniques) and were (iv) peer reviewed primary research, pre/post design with full texts in English. The included articles were selected and agreed upon by SO'B and LB.

### Data Extraction, Quality Assessment, and Analysis

The following data were extracted from each study: number of participants included, participant characteristics (demographic, age, gender, CP type, topographical description, Gross Motor Function Classification Scale (GMFCS) and medication use), intervention protocol (type, length, frequency, session duration, total sessions, initial prescription and progression, equipment used, training location, supervision and adherence), neurophysiological outcome measures (type, muscles tested and the activity used during measurement), functional outcome measures and study results. Studies were assessed for methodological quality by two raters (SO'B and LB) using the Downs and Black (26) quality assessment tool which assesses quality of reporting, internal and external validity, and power across five sub-scales, providing a total score out of 32, and are presented in Table 1. Outcome means and standard deviations were extracted to calculate effect sizes (Mean_post_-Mean_pre_/SD_pooled_) with 95% confidence intervals. An effect size of ≤0.20 was considered a trivial effect, 0.20 a small effect size, 0.50 a moderate effect size, and ≥0.80 a large effect size (36).

**Table 1:** Downs and Black methodological quality assessment tool.

**Study**	**Reporting/11**	**External validity/3**	**Bias/7**	**Confounders/6**	**Power/5**	**TOTAL/32**
Bleyenheuft et al. (27)	9	3	6	4	5	27
Colborne et al. (28)	4	1	4	1	0	10
Hodapp et al. (29)	7	1	4	2	0	14
Kurz et al. (30)	7	1	4	1	0	13
Olsen et al. (31)	8	1	3	1	0	13
Parvin et al. (32)	5	1	4	1	0	11
Phillips et al. (33)	9	2	3	1	0	15
Schalow et al. (34)	1	1	0	0	0	2
Willerslev-Oslen et al. (35)	6	1	4	1	0	12

## Results

The electronic database search yielded 6,263 potentially relevant studies. Following duplicate removal and initial screening of title and abstract against the inclusion criteria, full texts of the 71 remaining studies were scrutinized, resulting in nine eligible studies included in this review (Figure 1).

### Qualitative Assessment

The methodological quality of included studies is presented in Table 1. Overall methodological quality was very poor. The Downs and Black scores ranged from 2 to 27 out of a possible 32 points. All studies except one randomized control trial scored lowest on the external validity and power criteria.

### Study Design

Study design is presented in Table 2. There were three cohort studies (29, 33, 34), one cohort study with own control (35), one case-control study (32), one two period cross-over study (28), and two case reports (30, 31). There was one randomized control trial that measured neuromuscular control following lower limb training, however due to ongoing randomization and limited funding the groups were unbalanced (27).

**Table 2:** Study design and participant characteristics.

**Study**	**Study design**	**Summary (*n*, age range)**	**Group**	**Subjects *(n)***	**Mean age (range)**	**Sex (M/F)**	**CP Type**	**Topographical description**			**GMFCS**				**Medication use *(n)***
								**Hemi**	**Di**	**Quad**	**I**	**II**	**III**	**IV**	
Bleyenheuft et al. (27)	Randomized control trial*^a^*	41, NR	I	23	9.0 (NR)	12/11	NR	22	1	0	NR	NR	NR	NR	NR
			C	18	8.9 (NR)	9/9		18	0	0	NR	NR	NR	NR	NR
Colborne et al. (28)	Two period cross over	7, 8–15	I	7	10.57 (8–15)	4/3	NR	7	0	0	NR	NR	NR	NR	NR
Hodapp et al. (29)	Cohort	7, 5.2–15	I	7	9.7 (5.2–15)	NR	spastic = 7	0	7	0	1	3	3	0	nil
Kurz et al. (30)	Case report	4, 11–16	I	4	13.7 (11–16)	3/1	spastic = 4	0	4	0	0	0	3	1^*^	NR
Olsen et al. (31)	Case report	3, 6–14	I	2	10 (6–14)	1/1	spastic = 2	0	2	0	1	0	1	0	NR
			C	1^Δ^	14 (n/a)	1/0	n/a	n/a	n/a	n/a	n/a	n/a	n/a	n/a	NR
Parvin et al. (32)	Small cohort	4, NR	I	3	10.19 (NR)	2/1	spastic = 4	4	0	0	NR	NR	NR	NR	NR
			C	1	4.5 (n/a)	0/1									
Phillips et al. (33)	Cohort	6, 6–14	I	6	10 (6–14)	4/2	spastic = 6	4	2	0	NR	NR	NR	NR	NR
Schalow et al. (34)	Cohort	8, 7–27	I	8	15 (7–27)	NR	NR	NR	NR	NR	NR	NR	NR	NR	NR
Willerslev-Oslen et al. (35)	Cohort with own control	16, 5–14	I	16	9.6 (5–14)	11/5	NR	12	4	0	6	6	4	0	NR

*NR, not reported; I, intervention group; C, control group; n/a, not applicable*.

* *Participant was able to use a wheeled walker and solid AFOs for community ambulation when necessary*.

Δ *Typically developed*.

a *Due to ongoing recruitment and funding the groups became unbalanced*.

### Participants

The participant's characteristics from each study are presented in Table 2. Total participant numbers ranged from 3 to 41, each with a greater proportion of males. Both adults and children were included, ages spanned 5–27 years. Studies that reported on CP type were all spastic and the topographical classification included hemiplegia (most common) and diplegia. GMFCS levels ranged from I-IV, and all individuals could ambulate independently. Four studies included a control group (27, 31, 32, 35). The first of these control groups did not complete the intervention (27), the second control group consisted of a single typically developed participant (31), the third control group consisted of a single participant who received traditional occupational therapy (32), and the fourth group consisted of the intervention participants who underwent a control period (35). One study reported that no participants were receiving concurrent pharmacological treatment during the period of investigation (29), and eight studies did not report on medication use at the time of the intervention. No studies reported whether participants were receiving concurrent physical therapies during the intervention, and six studies did not report the participant's treatment history (27–29, 32, 32, 34, 35). Three studies reported treatment history including surgery and botulinum toxin injections which had occurred prior to an exclusion period (30, 31, 33).

### Interventions

A detailed description of intervention characteristics of the included studies is shown in Table 3. Six studies used treadmill walking (28–30, 32, 33, 35), two with the addition of body weight support through a harness system, one with the assistance of anti-gravity technology, and one with the addition of EMG biofeedback. One study implemented resistance training which only targeted the plantar flexors (31). One study used hand-and-arm-bimanual-intensive-therapy-including-lower-extremity (HABIT-ILE) (27). Coordinated dynamics therapy and physical therapy were each used in one study. Of note, Colborne et al. (28) used two interventions (walking plus biofeedback and physical therapy) in a cross over design. Thus, the current literature comprises a total of 11 different interventions. Intervention length varied from 10 days to 12 weeks and the range of session frequencies employed resulted in the total number of training sessions ranging from eight to 36. Training duration ranged from as little as 10 min up to 1 h. Treadmill speed and incline were commonly progressed throughout the training period, but this was not systematic (Table 3). Progression of the resistance training intervention was also adjusted as required. Neither the physical therapy nor coordinated dynamics therapy interventions reported on initial prescription or the rate of progression. One study reported upon training location, which was at home (35). The training equipment was most often highly specialized (e.g., hydraulic weight support system, coordinated dynamic therapy device, antigravity treadmill, dynamometer, and custom built dorsiflexion machine).

**Table 3:** Description of active movement training interventions.

**Study**	**Intervention type**	**Program**	**Intervention length**	**Frequency**	**Duration**	**Total sessions**	**Initial prescription**	**Progression**	**Training equipment**
Bleyenheuft et al. (27)	HABIT-ILE	UE: Gross dexterity, manipulative games and tasks, functional tasks, arts and craft, virtual reality. LE: Ball sitting, standing, balance board, virtual reality, walking/running, jumping, cycling, making scooter.	10 days	NR	90hrs total	NR	Activities selected on the basis of the child's motor abilities, age and interests. ~50% of time on bimanual activities requiring trunk and LE postural adaptations, 30% of time devoted to activities of daily living where standing and walking are required, and 20% of time spent in gross motor physical activities/play.	Progression of difficulty depended on success at the current level, usually after 3–5 successful tasks (of a repetitive task). More demanding activities were increasingly introduced.	Various: exercise equipment (e.g., fitness ball, balance board), active play equipment (e.g., jump rope, parachute) and electronic devices (e.g., Wii-fit).
Colborne et al. (28)	Group A: physical therapy	Therapy focused on isolated and controlled use of PF	4 weeks	2 days/week	45–60mins	8	NR	NR	NR
	Group B: treadmill walking+ EMGBF	Walking with concurrent visual and auditory EMG feedback	4 weeks	2 days/week	NR	8	NR	NR	CAF EMG feedback system
Hodapp et al. (29)	Treadmill training	Walking at a speed chosen to generate a regular gait pattern	10 consecutive days	7 days/week	10mins	10	Comfortable walking speed using a constant step length	Walking speed serially ↑ so the patient always felt comfortable	Treadmill
Kurz et al. (30)	BWSTT	Body weight supported walking	6 weeks	2 days/week	30mins	12	90% of over ground walking speed	Speed gradually ↑ (depending on ability to control stepping pattern) to ↑ number of steps practiced. Bodyweight support was manipulated to maintain upright lower limb posture, push off and toe clearance	Motorized treadmill with overhead available harness system (Litegait) and heart rate monitor
Olsen et al. (31)	PF strengthening	Concentric and eccentric PF at 30 and 90deg/s	12 weeks	3 days/week	~45min	36	Load set to ensure >80% of the maximum torque (tested at the beginning of the session) was being achieved	Load adjusted at each training as per ‘initial prescription’	Isokinetic KinCom dynamometer
Parvin et al. (32)	I: anti-gravity treadmill training	Anti-gravity treadmill walking	8 weeks	3 days/week	45 min	24	50% body weight supported at 1.5 km/hr	Body weight and speed was gradually increased in correspondence with the patients ability	AlterG treadmill
	C: occupational therapy	Traditional occupational therapy	8 weeks	3 days/week	NR	24	NR	NR	NR
Phillips et al. (33)	BWSTT	Body weight supported walking	2 weeks	6 days/week, twice daily	30 mins total (3 × 10 min, 5 min rest)	24	30% bodyweight assisted, initial speed (range): 2.4–3.1km/h	Bodyweight support reduced from 30 to 0% by the end of training. Treadmill speed increased to 3.7–5.0 km/h with training	Motorized treadmill with variable speed control and hydraulic weight support system (Litegait)
Schalow et al. (34)	Coordinated dynamics therapy	Use of CDT device	12 weeks	NR	4 h/week (48 h total)	NR	NR	NR	CDT device
Willerslev-Oslen et al. (35)	Gait training	Walking, ensuring heel contact in early stance	4 weeks	7 days/week	30 min total (smaller bouts allowed)	28	Speed and incline based on settings selected at first testing session	Incline and speed increased as exercise tolerance improved, parents encouraged progression	Treadmill (provided in home)

*HABIT-ILE, hand-and-arm-bimanual-intensive-therapy-including-lower-extremity; UE, upper extremity; LE, lower extremity; NR, not reported; PF, plantar flexors; EMGBF, electromyography biofeedback; BWSTT, body weight supported treadmill training; CDT, coordinated dynamics therapy; ↑, increase; I, intervention group; C, control group*.

### Adherence and Supervision

Adherence was only reported in one study, at 100% compliance with all sessions (30). One study required the therapist to document what was completed in each session in a logbook (28). One study provided parents with a diary to record the duration and specific activity performed during training sessions, factors preventing training, other physical activity performed that day and rate how the child felt during each session (35). Exercise sessions were supervised by trainers (29, 32), physical therapists (28, 30, 31, 33, 34) and parents or family members (34, 35).

### Neurophysiological Outcome Measures

Six studies reported on a total of 16 different electrophysiological outcome measures which are presented in Table 4. Electromyography was recorded from different lower limb muscles (most frequently tibialis anterior) and during different tasks (sitting, standing, walking, dorsiflexion maximum voluntary contraction, Gross Motor Function Measure (GMFM) items, and coordinated dynamic therapy). The results of coordinated dynamic therapy were not presented for all seven subjects, raw EMG of one individual was discussed as an example (34). H-reflexes during the swing phase of gait, H-reflexes over the whole step cycle, and gamma band (35–65 Hz) coherence were the only neurophysiological outcome measures to change statistically significantly following active movement training.

**Table 4:** Neurophysiological outcome measures and results.

**Study**	**Neurophysiology measure type**	**Outcome measure**	**Muscle/s measured**	**Measured during**	**Unit of measure**	**Result**
Bleyenheuft et al. (27)	Neuroimaging	MRI (DTI) Fractional anisotropy (focal analysis and whole tract analysis)	n/a	Supine rest	NR	Focal analysis: CST-LH sig effect of time. Whole tract analysis: Significant effect of time and group x time interaction for CST-NLH; significantly increased post intervention. Significant effect of time and group x time interaction for CST-LH; significantly increased post intervention.
		MRI (DTI) Mean diffusivity (focal analysis and whole tract analysis)	n/a	Supine rest	NR	Focal analysis: Significant effect of time and group x time interaction; reduced following intervention. Whole tract analysis: Significant effect of time and group × time interaction for CST-NLH; reduced following intervention. A group x time interaction for CST-LH; reduced following intervention.
		MRI (DTI) Voxels of CST (focal analysis and whole tract analysis)	n/a	Supine rest	voxels	Focal analysis: Significant effect of time in CST-NLH.
Colborne et al. (28)	Electrophysiology	Ensemble-averaged EMG	Tibialis anterior, triceps surae, lateral quadriceps, and medial hamstrings	Treadmill walking	NR	PT: Sustained tibialis anterior activity through stance, small ↓ in triceps surae reflex burst.EMGBF: ↓ excess triceps surae activity further than PT, but produced some lingering activity in the quadriceps.
Hodapp et al. (29)	Electrophysiology	M-max amplitude	Soleus	Standing	mV	ns
		H/M ratio	Soleus	Standing	% of M-max	ns
		H-reflex amplitudes over the whole step cycle	Soleus	Treadmill walking	mV	↓^*^
		H-reflex amplitudes in swing phase	Soleus	Treadmill walking	mV	↓^*^
		H-reflex amplitudes in stance phase	Soleus	Treadmill walking	mV	ns
		Absolute background EMG	Soleus	Treadmill walking	mV	ns
Kurz et al. (30)	Neuroimaging	MEG	n/a	Seated unilateral tibial nerve stimulation	Femto-Teslas amplitude (fT)	Source amplitude: 24% ↓ for the left foot, 45% ↓ for the right foot
Olsen et al. (31)	Electrophysiology	Muscle activation on-off ratio	Gastrocnemius and tibialis anterior	GMFM items D and E	n/a	S1: ↓ gastrocnemius and tibialis anterior on-off ratios for 2/3 GMFM items.S2: variable changes depending on GMFM item, muscle and leg. On- off ratios showed considerable ↑ bilaterally.
Parvin et al. (32)	Electrophysiology	M-wave amplitude	Soleus	NR	% change	Improved. I (range): 22.5–195.9;C: −35.5
		H-reflex latency	Soleus	NR	% change	Improved. I (range): 1.1–4.8; C: –6.1
		MEP amplitude	Tibialis Anterior	Seated with relaxed legs and fixed head position	% change	MEP amplitude ↑
		MEP latency	Tibialis Anterior	As above	% change	Time of pulse propagation improved
		MEP cortical silent period	Tibialis Anterior	As above	% change	↑ cortical silent period
		MEP pulse amplitude	Tibialis Anterior	As above	% change	Training did not have a specific effect
Phillips et al. (33)	Neuroimaging	fMRI total activation in congenital middle cerebral artery stroke	n/a	Active DF of involved ankle	voxels	(*n* = 1) 46% ↑
		fMRI total activation in subcortical lesion	n/a	Active DF of involved ankle	voxels	(*n* = 2) 366% ↑ and 939% ↑
Schalow et al. (34)	Electrophysiology	EMG traces	Tibialis anterior and gastrocnemius	Coordinated dynamics therapy	n/a	Improvements of movements as quantified by sEMG through the quality of the motor programs were very small.
Willerslev-Oslen et al. (35)	Electrophysiology	EMG-EMG coherence of a single muscle.	Tibialis anterior	Treadmill walking	Hz	↑ coherence in the frequency range 10–50 Hz immediately post training. Significant ↑ in frequency band 15- 40 Hz. Coherence overall ↓ compared to healthy children and showed no clear ↑ with age.

*DTI, diffusion tensor imaging; CST-LH, corticospinal tract lesioned hemisphere; CST-NLH, corticospinal tract non lesioned hemisphere; PT, physical therapy; EMGBF, electromyography biofeedback; EMG, electromyography; MEG, magnetoencephalography; fMRI, functional magnetic resonance imaging; DF, dorsiflexion; GMFM, gross motor function measure; S, subject; ns, not significant; ↓, decrease; ↑, increase; MEP, motor evoked potential; NR, not reported; I, intervention group; C, control group*.

* *Statistically significant*.

### Neuroimaging Outcome Measures

Neuroimaging was used in three studies (27, 30, 33). Five neurophysiological outcome measures were reported (Table 4). Imaging techniques included functional magnetic resonance imaging (fMRI) (33), magnetoencephalography (MEG) (30) and diffusion tensor imaging (DTI), which were measured during different tasks (rest, active dorsiflexion and tibial nerve stimulation). Statistical analysis was not performed in two of these preliminary exploratory studies due to low participant numbers (30, 33). One study performed a two-way repeated measures ANOVA (27).

### Functional Outcome Measures

Functional outcome measures and results are presented in **Table 6**. One study did not measure any functional outcome measures (34). Parvin et al. (32) did not report functional outcome measures due to low participant numbers. No studies achieved the minimum detectable change (61.9, 64.0, and 47.4 m for GMFCS levels I, II, and III, respectively) for the 6 min walk test (37). There was a group x time effect of HABIT-ILE on the 6 min walk test (27). Overground and treadmill gait speed were the only functional outcome measure to improve significantly following active movement training (29, 33). A significant improvement in duration of swing, stance and both double stance phases is likely attributable to the significant increase in treadmill walking speed (29). Overground gait parameters of positive ankle work improved significantly following physical therapy, and peak ankle power significantly improved following treadmill training with EMG biofeedback (28).

### Quantitative Assessment

The effect sizes and confidence intervals for neurophysiological and functional outcome measures are summarized in Tables 5, 6. Due to low participant numbers, unavailable or insufficient data, an effect size could not be determined for all outcome measures. Studies with low participant numbers did not complete statistical analysis. The descriptive results for neurophysiological outcomes are presented in Table 4. Effect sizes for neurophysiological measures varied widely. Effect sizes among studies which used the same functional outcome measure ranged from trivial to large.

**Table 5:** Effect sizes for neurophysiological outcome measures.

**Study**	**Measure**	**Measurement unit**	***n***	**Mean difference**	**Effect size**	**95% CI (lower)**	**95% CI (upper)**	***p* value**
**A. Neuroimaging**								
Bleyeneheuft et al. (27)	Whole tract anistropy (LH)	NR	23	0.03	0.64	0.05	1.23	<0.001^a^
	Whole tract anistropy (NLH)	NR	23	0.04	0.65	0.06	1.25	0.049^a^
Kurz et al. (30)	Source Amplitude	nAM [left foot]	3	3.20	0.58	−1.06	2.21	-
		nAM [right foot]	3	8.30	1.16	−0.57	2.89	-
Phillips et al. (33)	Active volume	cm^3^	3	-	-	-	-	-
	Total fMRI activation	(^Δ^SV_a_)	3	-	-	-	-	-
**B. Electrophysiology**								
Colborne et al. (28)	Ensemble-averaged EMG	NR	7	-	-	-	-	-
Hodapp et al. (29)	M-max during standing	mV	7	0.03	0.00	−1.04	1.05	ns
	H/M ratio during standing	% of Mmax	7	1.40	0.03	−1.02	1.08	ns
	H-reflexes over the whole step cycle	% of Mmax	7	14.10	3.50	1.83	5.16	<0.05
	H-reflex amplitudes in swing phase	mV	7	11.80	4.13	2.28	5.99	<0.05
	H-reflex amplitudes in stance phase	mV	7	13.00	0.88	−0.21	1.98	0.40
	Absolute background EMG	mV	7	0.03	0.09	−0.96	1.14	0.50
Olsen et al. (31)	Muscle activation on-off ratio	n/a	2	-	-	-	-	-
Parvin et al. (32)	H-reflex latency^Δ^		4	-	-	-	-	-
	M-wave amplitude^Δ^		4	-	-	-	-	-
	MEP amplitude^Δ^		2	-	-	-	-	-
	MEP latency^Δ^		2	-	-	-	-	-
	MEP cortical silent period^Δ^		2	-	-	-	-	-
	MEP intensity^Δ^		2	-	-	-	-	-
Schalow et al. (34)	EMG traces	n/a	8	-	-	-	-	-
Willerslev-Oslen et al. (35)	Coherence	Alpha band (5–15 Hz): amount of coherence (logarithm of cumulated sum within frequency band)^*^	16	-	-	-	-	ns
		Beta band (15–35 Hz): amount of coherence (logarithm of cumulated sum within frequency band)^*^	16	-	-	-	-	ns
		Gamma band (35–65 Hz): amount of coherence (logarithm of cumulated sum within frequency band)^*^	16	-	-	-	-	<0.01

*LH, lesioned hemisphere; NLH, nonlesioned hemisphere; fMRI, functional magnetic resonance imaging; EMG, electromyography; SV_a_, product of mean blood oxygenation level dependent percentage signal change of voxels and volume of supra-threshold voxels; NR, not reported; CI, confidence interval; mV, millivolts; n/a, not applicable; nAM, source amplitude*.

* *Pooled estimates*.

Δ *Pre-post values not available*.

a *Group X time*.

**Table 6:** Effect sizes for functional outcome measures.

**Measure**	**Study**	**Measurement units [sub measure, group]**	***n***	**Mean difference**	**Effect size**	**95% CI (lower)**	**95% CI (upper)**	***p* value**
10m walk test	Kurz et al. (30)	s	4	3.80	0.37	−1.03	1.77	-
	Phillips et al. (33)	m/s	6	0.19	0.52	−0.63	1.67	0.035
6MWT	Bleyenheuft et al. (27)	m	23	49	0.81	0.21	1.41	0.011^a^
	Kurz et al. (30)	m	4	24.00	0.29	−1.10	1.69	-
	Phillips et al. (33)	m	6	7.00	0.08	−1.05	1.21	0.851
GMFM	Colborne et al. (28)	% [Dimension D, PT]	7	0.50	-	-	-	-
		% [Dimension D, EMGBF]	7	0.20	-	-	-	-
		% [Dimension E, PT]	7	2.10	-	-	-	-
		% [Dimension E, EMGBF]	7	2.80	-	-	-	-
		% [Total (D+E), PT]	7	1.30	-	-	-	-
		% [Total (D+E), EMGBF]	7	1.50	-	-	-	-
	Phillips et al. (33)	% [Dimension E]	6	2.67	-	-	-	0.072
	Olsen et al. (31)	% [Total GMFM]	2	1.30	-	-	-	-
Treadmill gait speed	Hodapp et al. (29)	km/h	7	0.86	0.84	−0.25	1.93	<0.05
	Kurz et al. (30)	m/s	4	0.36	8.32	4.01	12.63	-
	Willerslev-Oslen et al. (35)		16	0.90	1.00	0.27	1.74	-
Overground gait speed	Colborne et al. (28)	m/s [PT]	7	0.00	0.00	−1.05	1.05	ns
		m/s [EMGBF]	7	0.06	0.89	−0.21	1.99	ns
	Hodapp et al. (29)	km/h	7	0.35	0.23	−0.82	1.28	<0.05
Duration of swing phase (TM)	Hodapp et al. (29)	% of step duration	7	2.50	0.30	−0.75	1.35	<0.05
Duration of stance phase (TM)	Hodapp et al. (29)	% of step duration	7	2.30	0.28	−0.78	1.33	<0.05
Duration of double stance Phase I (TM)	Hodapp et al. (29)	% of step duration	7	1.50	0.25	−0.81	1.30	<0.05
Duration of double stance Phase II (TM)	Hodapp et al. (29)	% of step duration	7	3.40	0.47	−0.59	1.53	<0.05
Stride length (OG)	Colborne et al. (28)	m [PT]	7	0.00	0.00	−1.05	1.05	ns
		m [EMGBF]	7	0.07	1.04	−0.08	2.15	ns
Stride time (OG)	Colborne et al. (28)	s [PT]	7	0.04	0.76	−0.33	1.84	ns
		s [EMGBF]	7	0.00	0.00	−1.05	1.05	ns
Stance/swing time (OG)	Colborne et al. (28)	- [PT]	7	0.10	0.94	−0.16	2.05	ns
		- [EMGBF]	7	0.03	0.32	−0.73	1.38	ns
Ankle DF in stance (OG)	Colborne et al. (28)	deg [PT]	7	2.50	1.02	−0.09	2.14	ns
		deg [EMGBF]	7	0.00	0.00	−1.05	1.05	ns
Ankle AROM (OG)	Colborne et al. (28)	deg [PT]	7	3.20	0.57	−0.50	1.64	ns
		deg [EMGBF]	7	1.40	0.25	−0.80	1.30	ns
Positive work at the hip (OG)	Colborne et al. (28)	J/kg [PT]	7	0.03	0.57	−0.50	1.64	ns
		J/kg [EMGBF]	7	0.05	0.94	−0.16	2.05	ns
Positive work at the ankle (OG)	Colborne et al. (28)	J/kg [PT]	7	0.04	1.51	0.32	2.70	0.05
		J/kg [EMGBF]	7	0.02	0.76	−0.33	1.84	ns
Negative work at the ankle (OG)	Colborne et al. (28)	J/kg [PT]	7	0.03	0.44	−0.62	1.51	ns
		J/kg [EMGBF]	7	0.02	0.30	−0.76	1.35	ns
Total positive work, hip and ankle (OG)	Colborne et al. (28)	J/kg [PT]	7	0.01	0.13	−0.92	1.17	ns
		J/kg [EMGBF]	7	0.07	1.04	−0.08	2.15	ns
Peak power generation at the ankle (OG)	Colborne et al. (28)	W/kg [PT]	7	0.25	0.76	−0.33	1.84	ns
		W/kg [EMGBF]	7	0.35	1.05	−0.07	2.17	0.1

*6MWT, six minute walk test; PT, physical therapy; EMGBF, electromyography biofeedback; CI, confidence interval; GMFM, gross motor function measure; TM, treadmill; OG, over ground; AROM, active range of motion*.

a *Group X time*.

## Discussion

The purpose of this study was to systematically review the current literature to determine the impact of lower limb active movement training on neuromuscular control in CP. Nine studies investigating neurophysiological outcomes of active movement training in individuals with CP were included in this review. Improved cortical representation of the ankle and reduced reflex amplitudes during gait allude to a potential for active movement training interventions to elicit favorable lower limb neuromuscular changes in CP. However, the mechanism/s for improvement are unable to be established due to a limited number of studies and diversity of outcome measures used. No study reported deterioration of outcome measures following interventions.

Treadmill training was the most common intervention. Six of the nine studies incorporated variations of treadmill walking training with a training dose ranging from 1.6 h (across 10 days) to 18 h (across 8 weeks). Following body weight supported treadmill training, favorable central nervous system adaptations for motor skill learning were reported. These include fMRI data suggestive of an increase in the area of the primary motor cortex active during isolated voluntary ankle movement (33), and a more refined representation of the foot in the somatosensory cortices, as identified using magnetoencephalography (MEG) during peripheral nerve stimulation (30). One study quantified changes in corticospinal tract (CST) integrity using diffusion tensor imaging (DTI) following a combined upper and lower limb training protocol (27). There was a significant positive group x time effect of training for the whole tract analyses, and lower limb function quantified using a 6 min walk test. Correlations between focal analyses of CST integrity and hand motor function showed a trend for improvement in hand function, but had no correlation to lower limb performance on the 6 min walk test. Combined upper and lower limb intensive motor training appears to have a favorable impact on CST integrity and hand function. However, it is unclear whether or not lower limb training contributed to the improvements in CST integrity observed in this intervention. Although there was a trend toward a relationship between improved CST changes and hand, but not leg, motor function, this observation does not rule out an effect of lower limb training on CSTs. Any such relationship between CST integrity and motor function may have been biased toward the upper limb due to a larger number of upper limb CST axons in the area of measurement. There was also only one physical lower limb performance assessment measure, which may not have been representative of functions affected strongly by CST integrity. It therefore remains unclear what effect lower limb training has on CST integrity following lower limb motor skill learning.

Favorable peripheral nervous system changes were reported in four gait training studies from varied outcome measures which overall indicate a more functionally useful swing phase and foot placement during gait. Improvements in dorsiflexion prior to foot contact and reflex modulation (reduced soleus H-reflex amplitude) (29), improved tibialis anterior coordination enhancing toe lift (coherence between proximal and distal ends of tibialis anterior of the most affected leg) (35), and triceps surae and tibialis anterior activation patterns (sEMG) more similar to typically developed children were reported (28). The fourth small cohort study (*n* = 2) reported combined changes in the central and peripheral nervous system, with improved corticospinal tract connectivity [increased motor evoked potential (MEP) amplitude during transcranial magnetic stimulation (TMS)] and reduced inhibition of the tibial nerve (improved H-reflex latency and maximum M-wave amplitude), such that the measures approached values observed in typically developed individuals (32). The impact of training volume and progression on these outcomes is unclear as favorable changes were obtained following a range of 100–840 min of gait training (held across 10–28 sessions), and progression of difficulty was not based on categorical performance criteria being met and poorly described (Table 3). In summary, although there are reports of improved neuromuscular control of the ankle plantar flexors and dorsiflexors during gait following treadmill training, the lack of consistency in training doses and objective outcome measures makes it difficult to make recommendations for interventions and monitoring changes.

Similarly, following coordinated dynamics therapy (crawling, treadmill walking, jumping on a spring-board and use of a coordination dynamic therapy device resembling a combined arm and leg ergometer performed seated or supine), sEMG was reported (through visual inspection) to be changed in a favorable way, but this remained unquantified (34). Olsen et al. (31) demonstrated the feasibility of recording dorsi- and plantar flexion muscle activation patterns during selected GMFM items but was unable to determine whether changes in muscle activation sequencing (sEMG on-off ratio) were favorably improved following resistance training due to outcome variability in the small sample. Resistance training has been shown to increase muscle size and strength in individuals with CP (21, 38). Twelve weeks of lower limb, explosive, progressive, heavy resistance training has recently been shown to increase lower limb muscular strength and rate of force development in tibialis anterior (39). Rate of force development is a measure of torque output during a rapid maximum muscle contraction, but is considered by some as a proxy neurophysiological measure because it is strongly determined by neural drive (40). The increase in rate of force development observed by Kirk et al. (39) was accompanied by improved maximum isometric voluntary dorsiflexion, plantar flexion, knee flexion, and knee extension strength. While it appears that strength training may have provided some improvement in capacity to activate the lower limb musculature in adults with CP, the exact mechanisms (neural vs. muscle adaptation) cannot be determined or quantified by these measures alone.

There have been numerous attempts to understand neuroplasticity and neuromuscular changes following interventions targeting muscular adaptations in humans (41–43), yet there is limited information pertaining to the trainability and measurement of neuromuscular control of the lower limb in CP. Of the nine studies included in this review, no studies performed the same neuromuscular outcome measure under the same conditions. This may have been limited by access to equipment and expertise required to perform such assessments. The location of adaptation within the nervous system following intervention is also unknown. It is particularly difficult to identify site specific changes within the nervous system as the results of non-intrusive neurophysiological measurements are often subject to modulation at multiple neural sites.

Although some nervous system changes were reported following active movement training, only three studies (that incorporated treadmill training and HABIT-ILE) found concomitant functional capacity improvements (27, 29, 33). Functional improvements in self-selected overground walking speed were elicited by a short walking training intervention (100 min across 10 days) (29). A slightly longer training duration of bodyweight supported treadmill walking (720 min across 2 weeks) improved walking speed during the 10 m walk test (33). These relatively short training periods suggest there may be an early adaptation period related to the specificity of the task of walking which improves neuromuscular control. These studies did not report on participant's current physical activity levels, familiarization and confidence with treadmill walking prior to the study.

With few studies measuring both neurophysiology and gross motor function outcomes following active movement training, we are not currently able to predict the impact and relationship of all neuromuscular changes on gross motor function. Further, the sensitivity of functional outcome measures to reflect neural changes also remains unknown. For example, intervention studies have demonstrated improvements in lower limb functional capacity without concurrent neurophysiological measurements (19, 38, 44), which limits our capacity to understand whether central and/or peripheral nervous system changes occur and contribute to these observed functional outcomes.

It is premature to speculate on the recommendations for optimal prescription of active movement training to elicit neuromuscular adaptation, due to the variability of interventions among studies and lack of consistency in choice of neuromuscular outcome measures. Despite treadmill training being a popular training mode, previous systematic reviews in children with CP have not been able to determine optimal protocol parameters (45, 46). Treadmill training is a safe and feasible training intervention, but the magnitude of benefit and improvement in lower limb neuromuscular control for individuals at different GMFCS levels is not clear. Large scale trials are still required before guidelines can be considered.

Individual study results must be interpreted with caution due to low study methodological quality, varied training loads and modalities, lack of control groups and diverse use of neurophysiological and neuroimaging outcome measures. Low participant numbers in small cohort studies do not provide sufficient power or external validity to establish whether changes in neuromuscular control are genuine. The effect of age on motor learning and whether there is an optimal time for neural plasticity is unknown. The large range in age of participants makes it difficult to understand both the age dependent adaptability of the nervous system, and the impact of development. Finally, as all participants in the studies reviewed here were diagnosed with spastic CP, and due to the differential effects of lesion location, the findings of nervous system changes suggested may not be generalisable to other types of CP.

## Future Direction

Future research should be conscious of the existing neurophysiological outcome measures used to evaluate training interventions, in order to allow for comparison of training mode and training load on the magnitude of change. Future work should also endeavor to apply greater methodological quality than the existing studies. Careful selection of outcome measures which adequately assess regions of the nervous system targeted by training (cortical, spinal cord, neuromuscular junction) is necessary to draw conclusions regarding the impact of training. No single measure will quantify the adaptability of the nervous system as a whole, but adequate and repeated attempts are required to determine mechanisms underlying motor control changes that may be important for function. Due to the complexity of the motor control system, measures that are sensitive enough to measure change over time are required. There are inherent difficulties with performing appropriate neurophysiological assessments. Equipment, expertise and funds are often not readily accessible to clinicians in clinics where these types of interventions and treatment plans are typically conducted.

## Conclusions

The impact of active movement training on lower limb neuromuscular control in individuals with CP cannot yet be established. Due to the small number of investigations and their low scientific quality, it is not possible to determine the mechanisms by which the different active movement interventions elicit change within the nervous system. There is disparity in the choice of outcome measures used between studies, which prevents direct comparisons between interventions and the identification of central vs. peripheral nervous system adaptations. Ultimately, the question of whether modalities such as strength training or gait training can favorably alter neuromuscular control of the lower limb in CP remains unclear and requires further investigation. It also remains unclear how changes in neurophysiological measures relate to changes or improvement in gross motor function in CP.

## Data Availability Statement

The datasets presented in this study can be found in online repositories. The names of the repositories and references can be found in the article.

## Author Contributions

SO'B, LB, GL, and TC: conceptualization and data interpretation. SO'B and LB: search and data extraction. SO'B: writing. LB, GL, and TC: reviewing and editing. All authors listed, have made substantial, direct and intellectual contribution to the work, and approved it for publication.

## Conflict of Interest

The authors declare that the research was conducted in the absence of any commercial or financial relationships that could be construed as a potential conflict of interest.
